# Summary of taxonomy changes ratified by the International Committee on Taxonomy of Viruses (ICTV) from the Fungal and Protist Viruses Subcommittee, 2025

**DOI:** 10.1099/jgv.0.002115

**Published:** 2025-07-25

**Authors:** Sead Sabanadzovic, Chantal Abergel, María A. Ayllón, Leticia Botella, Marta Canuti, Yuto Chiba, JeanMichel Claverie, Robert H.A. Coutts, Stefania Daghino, Livia Donaire, Marco Forgia, Ondřej Hejna, Jichun Jia, Daohong Jiang, Ioly Kotta-Loizou, Mart Krupovic, Andrew S. Lang, Matthieu Legendre, Shin-Yi Lee Marzano, Fan Mu, Uri Neri, Luca Nerva, Judit Pénzes, Anna Poimala, Sofia Rigou, Yukiyo Sato, Wajeeha Shamsi, Suvi Sutela, Nobuhiro Suzuki, Massimo Turina, Syun-ichi Urayama, Eeva J. Vainio, Jiatao Xie, ICTV Taxonomy Summary Consortium, E.M. Adriaenssens

**Affiliations:** 1 Department of Agricultural Science and Plant Protection, Mississippi State University, Mississippi, USA; 2 Institute for Genomics, Biocomputing and Biotechnology, Mississippi State University, Mississippi, USA; 3 Information Génomique & Structurale, UMR7256, CNRS & Aix-Marseille Université, Marseille, IMM, IM2B, IOM, Marseille, France; 4 Centro de Biotecnología y Genómica de Plantas, Universidad Politécnica de Madrid (UPM)—Instituto Nacional de Investigación y Tecnología Agraria y Alimentaria (INIA/CSIC), Campus de Montegancedo, Pozuelo de Alarcón, Madrid, Spain; 5 Departamento de Biotecnología-Biología Vegetal, Escuela Técnica Superior de Ingeniería Agronómica, Alimentaria y de Biosistemas, Universidad Politécnica de Madrid (UPM), Madrid, Spain; 6 Forest Protection and Wildlife Management Mendel University in Brno, Brno, Czechia; 7 Department of Veterinary and Animal Sciences, University of Copenhagen, Frederiksberg, Denmark; 8 School of Agriculture, Meiji University, Kawasaki, Japan; 9 School of Health, Medicine and Life Sciences, University of Hertfordshire, Hatfield, UK; 10 Institute for Sustainable Plant Protection, National Research Council of Italy, Torino, Italy; 11 Centro de Edafología y Biología Aplicada del Segura-CSIC, 30100 Murcia, Spain; 12 Institute for Sustainable Plant Protection, CNR, Bari, Italy; 13 Department of Genetics and Biotechnologies, University of South Bohemia, České Budějovice, Czechia; 14 College of Plant Protection, Shanxi Agricultural University, Jinzhong, Shanxi, PR China; 15 College of Plant Science and Technology, Huazhong Agricultural University, Wuhan, PR China; 16 Institut Pasteur, Université Paris Cité, CNRS UMR6047, Archaeal Virology Unit, Paris, France; 17 Department of Biology, Memorial University of Newfoundland, St. John’s, Canada; 18 United States Department of Agriculture, Agricultural Research Service, Application Technology Research Unit, Toledo, Ohio, USA; 19 The Shmunis School of Biomedicine and Cancer Research, Tel Aviv University, Israel; 20 Council for Agricultural Research and Economics - Research Centre for Viticulture and Enology, Roma, Italy; 21 Department of Entomology, Texas A&M University, College Station, TX, USA; 22 Natural Resources Institute Finland (Luke), Helsinki, Finland; 23 Department of Biology, Institute for Plant Sciences, University of Cologne, Cologne, Germany; 24 Department of Molecular Biology and Genetics, Aarhus University, 8000 Aarhus C, Denmark; 25 Institute of Plant Science and Resources, Okayama University, Kurashiki, Japan; 26 Institute for Sustainable Plant Protection-URT Brescia, National Research Council of Italy, 25123, Brescia, Italy; 27 Department of Plant Protection, School of Agriculture, The University of Jordan, Amman, 11942, Jordan; 28 Department of Life and Environmental Sciences, University of Tsukuba, Tsukuba, Japan

**Keywords:** *Acremonium magoulivirus*, *Alphacedratvirus aljazairense*, *Alphacedratvirus aljazairmassiliense*, *Alphacedratvirus franciense*, *Alphacedratvirus francolausannense*, *Alphahydrivirus*, *Alphahydrivirus permafrostis*, *Alphaormycoviridae*, *Alphasclernavirus*, *Alphasclernavirus alphasclerotiniae*, *Alphasclernavirus betasclerotiniae*, *Aspergillus penoulivirus*, *Astarnavirus*, *Aurantiochytrium *single-stranded RNA virus 01, *Avoomyvirus*, *Avoomyvirus amagae*, *Avoomyvirus chiwundudzae*, *Avoomyvirus homensis*, *Avoomyvirus ibuensis*, *Avoomyvirus kabensis*, *Avoomyvirus kapangae*, *Avoomyvirus kifensis*, *Avoomyvirus kulapukae*, *Avoomyvirus mantarae*, *Avoomyvirus meijunae*, *Avoomyvirus mocensis*, *Avoomyvirus moengjensis*, *Avoomyvirus mosidurae*, *Avoomyvirus mouchlis*, *Avoomyvirus mucegae*, *Avoomyvirus myglensis*, *Avoomyvirus qurwarae*, *Avoomyvirus tavemmaltae*, *Avoomyvirus tayumensis*, *Avoomyvirus tchamosseurae*, *Avoomyvirus tewensis*, *Bacillarnavirus nagasakii*, *Bacillarnavirus setoensis*, *Bacillarnavirus yujii*, *Betaormycoviridae*, *Betasclernavirus*, *Betasclernavirus alphafusarii*, *Betasclernavirus alphasclerotii*, *Betasclernavirus betafusarii*, *Betasclernavirus betasclerotii*, *Betasclernavirus botrytidis*, *Bormycovirales*, *Bormycovirus*, *Bormycovirus duphytophthorae*, *Bormycovirus trephytophthorae*, *Bormycovirus unphytophthorae*, *Bormycovirus verticilli*, *Botoulivirus alphasclerotiniae*, *Botoulivirus betasclerotiniae*, *Botoulivirus botrytidis*, *Botoulivirus epicocci*, *Botrytis botoulivirus*, *Britarnavirus 1*, *Britarnavirus 2*, *Britarnavirus 3*, *Britarnavirus 4*, *Cassava virus C*, *Chaetarnavirus 2*, *Chaetenuissarnavirus II*, *Chaetoceros socialis forma radians RNA virus 1*, *Chaetoceros tenuissimus RNA virus 01*, *Cladosporium magoulivirus 1*, *Cladosporium magoulivirus 2*, *Cladosporium penoulivirus*, *Cladosporium scleroulivirus*, *Colletotrichum magoulivirus*, *Delepalmivirus*, *Delepalmivirus ibericum*, *Delepalmivirus magnaporthae*, *Delepalmivirus oidiodendri*, *Delepalmivirus sclerotiniae*, *Deltanormycoviridae*, *Divipalmivirus*, *Divipalmivirus aspergilli*, *Divipalmivirus cryphonectriae*, *Divipalmivirus diplodiae*, *Divipalmivirus italiense*, *Divipalmivirus japonicum*, *Divipalmivirus suilli*, *Dormycovirus*, *Dormycovirus erysiphe*, *Dormycovirus phytophthorae*, *Dormycovirus plasmoparae*, *Epicoccum botoulivirus*, *Epicoccum penoulivirus*, *Epirus cherry virus*, *Formycovirales*, *Gammaormycoviridae*, *Heterosigma akashiwo RNA virus*, *Hormycovirus*, *Hormycovirus hortiboleti*, *Hydriviridae*, *Jakapalmivirus*, *Jakapalmivirus botritidis*, *Jakapalmivirus bremiae*, *Jakapalmivirus cinereae*, *Jakapalmivirus ibericum*, *Jakapalmivirus italiense*, *Jakapalmivirus sclerotiniae*, *Jericarnavirus A*, *Jericarnavirus B*, *Kusarnavirus tomaruii*, *Labyrnavirus takaoii*, *Lineavirales*, *Locarnavirus derisii*, *Locarnavirus greningerii*, *Locarnavirus jerichoensis*, *Locarnavirus rohweri*, *Magnaporthe magoulivirus 1*, *Magnaporthe penoulivirus*, *Magoulivirus acremonii*, *Magoulivirus cladosporii*, *Magoulivirus colletotrichi*, *Magoulivirus oryzae*, *Magoulivirus penicillii*, *Magoulivirus phaeoacremonii*, *Magoulivirus plasmoparae*, *Magoulivirus rhizoctoniae*, *Marnavirus taichanarum*, *Mycoalphaviridae*, *Neofusicoccum penoulivirus*, *Nicoomyvirus*, *Nicoomyvirus beschimmelingae*, *Nicoomyvirus bolorensis*, *Nicoomyvirus floridurae*, *Nicoomyvirus hallitusensis*, *Nicoomyvirus lizumae*, *Nicoomyvirus llwydnae*, *Nicoomyvirus moegelae*, *Nicoomyvirus mohonsis*, *Nicoomyvirus moisissurensis*, *Nicoomyvirus moldensis*, *Nicoomyvirus muffae*, *Nicoomyvirus peneszensis*, *Nicoomyvirus plesenae*, *Nicoomyvirus schimmelae*, *Nicoomyvirus simensis*, *Nicoomyvirus svampae*, *Ocovirineae*, *Oomyviridae*, *Orpoviricetes*, *Orthocedratvirinae*, *Orthopithovirinae*, *Ourmia melon virus*, *Ourmiavirus cucurbitae*, *Ourmiavirus manihoti*, *Ourmiavirus pruni*, *Palmarnavirus 128*, *Palmarnavirus 156*, *Palmarnavirus 473*, *Penicillium magoulivirus*, *Penicillium penoulivirus*, *Penoulivirus aspergilli*, *Penoulivirus cladosporii*, *Penoulivirus epicocci*, *Penoulivirus neofusicocci*, *Penoulivirus oryzae*, *Penoulivirus penicillii*, *Penoulivirus phaeoacremonii*, *Penoulivirus phomae*, *Penoulivirus phomopsis*, *Penoulivirus pyriculariae*, *Penoulivirus sclerotiniae*, *Phaeoacremonium magoulivirus*, *Phaeoacremonium penoulivirus*, *Phoma penoulivirus*, *Phomosis penoulivirus*, *Phormycovirus*, *Phormycovirus duphytophthorae*, *Phormycovirus phytophthorae*, *Phormycovirus plasmoparae*, *Phormycovirus quaphytophthorae*, *Phormycovirus trephytophthorae*, *Phormycovirus unphytophthorae*, *Pimascovirales*, *Pyricularia penoulivirus*, *Pyricularia scleroulivirus 3*, *Rhizoctonia magoulivirus 1*, *Rhizoctonia rhizoulivirus*, *Rhizosolenia setigera RNA virus 01*, *Rhizoulivirus rhizoctoniae*, *Salisharnavirus britensis*, *Salisharnavirus mirandaeae*, *Salisharnavirus stewardii*, *Salisharnavirus vlokiae*, *Sanfarnavirus 1*, *Sanfarnavirus 2*, *Sanfarnavirus 3*, *Sclerotinia botoulivirus 2*, *Sclerotinia botoulivirus 3*, *Sclerotinia penoulivirus*, *Sclerotinia scleroulivirus 1*, *Scleroulivirus alphaglycinae*, *Scleroulivirus betaglycinae*, *Scleroulivirus cladosporii*, *Scleroulivirus pyriculariae*, *Scleroulivirus sclerotiniae*, *Sogarnavirus culleyi*, *Sogarnavirus gustavseniae*, *Sogarnavirus kimuraei*, *Sogarnavirus kitsilanoensis*, *Sogarnavirus palmerensis*, *Sogarnavirus tomaruii*, *Soybean scleroulivirus 1*, *Soybean scleroulivirus 2*, *Splipalmiviridae*, *Stormycovirus*, *Stormycovirus alariae*, *Stormycovirus starmellariae*, *Swoomyvirus*, *Swoomyvirus plijesanensis*, *Tormycovirus*, *Tormycovirus duaplasmoparae*, *Tormycovirus erysiphe*, *Tormycovirus fusarii*, *Tormycovirus thrichodermae*, *Tormycovirus unplasmoparae*, *Unirnavirus*, *Unirnavirus aldianthicolae*, *Unirnavirus allongipei*, *Unirnavirus aspergilli*, *Unirnavirus beauveriae*, *Unirnavirus cogleosporioidei*, *Unirnavirus cohigginsiani*, *Unirnavirus combuense*, *Unirnavirus fusarii*, *Unirnavirus pripenicillii*, *Unirnavirus prustilaginoideae*, *Unirnavirus secupenicillii*, *Unirnavirus secustilaginoideae*, *Unirnavirus trichodermae*, *Vormycovirus*, *Vormycovirus duerysiphe*, *Vormycovirus ophiocordyceps*, *Vormycovirus plasmoparae*, *Vormycovirus unerysiphe*, *Vormycovirus verticilli*

## Abstract

*An erratum of this article has been published full details can be found at 10.1099/jgv.0.002144*

The Fungal and Protist Viruses Subcommittee (SC) of the International Committee on Taxonomy of Viruses (ICTV) has received a total of eight taxonomic proposals for the 2024 annual cycle. The extent of proposed changes varied, including nomenclatural updates, creation of new taxa and reorganization of established taxa. Following the ICTV procedures, all proposals were reviewed and voted upon by the members of the Executive Committee with ratification in March 2025. As a result, a total of 52 species in the families *Botourmiaviridae* and *Marnaviridae* were renamed to comply with the mandated binomial format. A new genus has been added to the dsRNA virus family *Amalgaviridae*, while two new families, *Splipalmiviridae* (*Wolframvirales*) and *Mycoalphaviridae* (*Hepelivirales*), were created to classify new groups of positive-sense (+) RNA mycoviruses. The class *Arfiviricetes* (*Cressdnaviricota*) was expanded by a new order *Lineavirales* and a new family *Oomyviridae* of ssDNA viruses. Additionally, a new class *Orpoviricetes* was created in the kingdom *Orthornavirae* to classify a group of bisegmented (+)RNA viruses reported from fungi and oomycetes. Finally, the order *Pimascovirales* was reorganized to better depict evolutionary relationships of pithoviruses and related viruses with large dsDNA genomes. The summary of updates in the taxonomy of fungal and protist viruses presented here is limited to taxa within the remit of this Subcommittee. For information on taxonomy changes on other fungal viruses closely related to animal and/or plant viruses, please see reports from sister ICTV Subcommittees (i.e. Plant Virus SC and Animal dsRNA and ssRNA(−) Viruses SC).

## Introduction

The Fungal and Protist Viruses Subcommittee (SC) of the International Committee on Taxonomy of Viruses (ICTV) is tasked with the refinement and development of the taxonomy of fungal and protist viruses. The SC has been recently reorganized to comprise 13 taxon-specific Study Groups (SG) composed of 60 experts from 18 different countries. SGs currently under the remit of this Subcommittee are formally organized at family (*Botourmiaviridae*, *Curvulaviridae*, *Fusariviridae*, *Hypoviridae*, *Marnaviridae*, *Partitiviridae* and *Pithoviridae*), order (*Algavirales*, *Ghabrivirales*, *Imitervirales* and *Wolframvirales*) or phylum (*Ambiviricota*) level. In addition, there is an ad hoc SG dealing with the taxonomy of ‘virophages’. However, the structure of this SC will undergo further changes, including the addition of new taxon-specific expert groups, to reflect our ever-expanding knowledge on the diversity of fungal and protist viruses.

The ICTV Fungal and Protist Viruses SC received a total of eight taxonomic proposals (TaxoProps) for the 2024 annual cycle of taxonomic updates. These proposals are a result of a collaborative effort by a total of 33 scientists, a third of whom are not officially involved in any of the current ICTV SGs. Following the standard ICTV procedures [1], the TaxoProps were reviewed and voted upon by the Executive Committee during the Annual Meeting (EC56) held in Bari (Italy) in August 2024, revised when needed and further scrutinized during the ratification voting period by all ICTV members in March 2025. All eight proposals were approved by a majority vote. Here, we briefly report on recent changes in the taxonomy of viruses belonging to taxa under the remit of this ICTV SC.

The extent of proposed changes varied among the different TaxoProps. Namely, the adoption of the binomial format for species names, mandated by the ICTV [2,3], was completed for the families *Botourmiaviridae* (total=32 species) [4] and *Marnaviridae* (total=20 species). In both cases, the Latinized binomial format was preferred over the ‘freeform’ option. The family *Amalgaviridae* was expanded by the creation of a third genus, *Unirnavirus*, containing 13 new species of dsRNA viruses with a non-segmented genome [5,6]. Furthermore, the order *Wolframvirales* was expanded with 1 new family, *Splipalmiviridae* (3 genera, 16 species), including a group of recently discovered viruses encoding a narnavirus-like RNA-directed RNA polymerase (RdRP) divided into two genomic segments [7,10]. Additionally, a new family *Mycoalphaviridae* (two genera, seven species) composed of positive-sense RNA viruses with an RdRP related to that of members of the order *Hepeliviriales* was created [11,12]. Concerning higher-rank taxa, a new order, *Lineavirales*, including 1 family, 3 genera and 38 species of viruses with linear ssDNA genomes [13,14], was included in the class *Arfiviricetes* of the phylum *Cressdnaviricota* [15]. Furthermore, a new ‘floating’ class *Orpoviricetes* (including 2 orders, 5 families, 7 genera and 26 species) in the kingdom *Orthornavirae* was created to classify a group of recently discovered bisegmented positive-sense RNA viruses of fungi and oomycetes characterized by highly diverged RdRPs [16,18]. Finally, the order *Pimascovirales*, after recent changes [19], was further reorganized to include several new taxa (one suborder, one family, one genus and one species), whereas the existing family *Pithoviridae* has been split into two subfamilies, and two alphacedratvirus species have been renamed. The revised taxonomy more adequately reflects the evolutionary relationships between the original pithovirus [20] and members of the families *Pithoviridae*, *Orpheoviridae* and *Hydriviridae* compared to viruses belonging to other families in the order.

For a complete update on the taxonomy of fungal and protist viruses, please also see reports from other ICTV SC, in particular, Plant Viruses SC [21] and Animal dsRNA and ssRNA(−) Viruses SC [22]. Final versions of all TaxoProps ratified in March 2025 are publicly available on the ICTV website (). A file including all the Tables of taxonomic changes below is available as a supplementary file to this article.

## Main Text

## Contents

2024.001F.Botourmiaviridae_spren
2024.002F.Marnaviridae_spren
2024.003F.Splipalmiviridae_newfam
2024.004F.Oomyviridae_newfam
2024.005F.Pimascovirales_reorg
2024.006F.Amalgaviridae_newgen
2024.007F.Mycoalphaviridae_newfam
2024.008F.Orpoviricetes_newclass

### 2024.001F.Botourmiaviridae_spren

**Title:** Change the name of 32 species of 6 genera of the family *Botourmiaviridae*

**Authors:** Ayllón MA (mariaangeles.ayllon@upm.es), Turina M, Donaire L, Nerva L, Marzano SYL, Xie J, Jiang D

**Summary**

**Taxonomic rank(s) affected**
Species
**Description of current taxonomy**
Species correctly classified inside the genus but with outdated names
**Proposed taxonomic change(s)**
We propose to change the name of 32 species in the genera *Botoulivirus*, *Magoulivirus*, *Ourmiavirus*, *Penoulivirus*, *Rhizoulivirus* and *Scleroulivirus* in the family *Botourmiaviridae.*
**Justification**
The names of 32 species in the genera *Botoulivirus*, *Magoulivirus*, *Ourmiavirus*, *Penoulivirus*, *Rhizoulivirus* and *Scleroulivirus* in the family *Botourmiaviridae* were not compliant with the binomial format, so in this proposal, we made changes to meet the ICTV criteria in naming species.
**Submitted**: 11/06/24

**Table 1.** *Botourmiaviridae*, 32 rename taxa*

**Table:** 

Operation	Rank	New taxon name	Previous taxon name
Rename taxon	Species	*Botoulivirus botrytidis*	*Botrytis botoulivirus*
Rename taxon	Species	*Botoulivirus epicocci*	*Epicoccum botoulivirus*
Rename taxon	Species	*Botoulivirus alphasclerotiniae*	*Sclerotinia botoulivirus 2*
Rename taxon	Species	*Botoulivirus betasclerotiniae*	*Sclerotinia botoulivirus 3*
Rename taxon	Species	*Magoulivirus acremonii*	*Acremonium magoulivirus*
Rename taxon	Species	*Magoulivirus plasmoparae*	*Cladosporium magoulivirus 1*
Rename taxon	Species	*Magoulivirus cladosporii*	*Cladosporium magoulivirus 2*
Rename taxon	Species	*Magoulivirus colletotrichi*	*Colletotrichum magoulivirus*
Rename taxon	Species	*Magoulivirus oryzae*	*Magnaporthe magoulivirus 1*
Rename taxon	Species	*Magoulivirus penicillii*	*Penicillium magoulivirus*
Rename taxon	Species	*Magoulivirus phaeoacremonii*	*Phaeoacremonium magoulivirus*
Rename taxon	Species	*Magoulivirus rhizoctoniae*	*Rhizoctonia magoulivirus 1*
Rename taxon	Species	*Ourmiavirus manihoti*	*Cassava virus C*
Rename taxon	Species	*Ourmiavirus pruni*	*Epirus cherry virus*
Rename taxon	Species	*Ourmiavirus cucurbitae*	*Ourmia melon virus*
Rename taxon	Species	*Penoulivirus aspergilli*	*Aspergillus penoulivirus*
Rename taxon	Species	*Penoulivirus cladosporii*	*Cladosporium penoulivirus*
Rename taxon	Species	*Penoulivirus epicocci*	*Epicoccum penoulivirus*
Rename taxon	Species	*Penoulivirus oryzae*	*Magnaporthe penoulivirus*
Rename taxon	Species	*Penoulivirus neofusicocci*	*Neofusicoccum penoulivirus*
Rename taxon	Species	*Penoulivirus penicillii*	*Penicillium penoulivirus*
Rename taxon	Species	*Penoulivirus phaeoacremonii*	*Phaeoacremonium penoulivirus*
Rename taxon	Species	*Penoulivirus phomae*	*Phoma penoulivirus*
Rename taxon	Species	*Penoulivirus phomopsis*	*Phomosis penoulivirus*
Rename taxon	Species	*Penoulivirus pyriculariae*	*Pyricularia penoulivirus*
Rename taxon	Species	*Penoulivirus sclerotiniae*	*Sclerotinia penoulivirus*
Rename taxon	Species	*Rhizoulivirus rhizoctoniae*	*Rhizoctonia rhizoulivirus*
Rename taxon	Species	*Scleroulivirus cladosporii*	*Cladosporium scleroulivirus*
Rename taxon	Species	*Scleroulivirus pyriculariae*	*Pyricularia scleroulivirus* 3
Rename taxon	Species	*Scleroulivirus sclerotiniae*	*Sclerotinia scleroulivirus* 1
Rename taxon	Species	*Scleroulivirus alphaglycinae*	*Soybean scleroulivirus* 1
Rename taxon	Species	*Scleroulivirus betaglycinae*	*Soybean scleroulivirus* 2

*Source/full text: https://ictv.global/ictv/proposals/2024.001F.Botourmiaviridae_spren.zip

### 2024.002F.Marnaviridae_spren

**Title:** Rename 20 species within family *Marnaviridae*

**Author:** Lang AS (aslang@mun.ca)

**Summary**

**Taxonomic rank(s) affected**
Species in the family.
**Description of current taxonomy**
The family *Marnaviridae* currently includes 7 genera and 20 species with inadequate nomenclature.
**Proposed taxonomic change(s)**
Changes in the names of all 20 currently classified species are proposed to adhere to newly adopted binomial nomenclatural standards/formats.
**Justification**
Proposed changes are required to comply with binomial species nomenclature mandated by the ICTV.
**Submitted**: 18/04/24; **Revised**: 17/10/24

**Table 2.** *Marnaviridae*, 20 rename taxa*

**Table:** 

Operation	Rank	New taxon name	Previous taxon name
Rename taxon	Species	*Bacillarnavirus yujii*	*Chaetoceros socialis forma radians RNA virus 1*
Rename taxon	Species	*Bacillarnavirus setoensis*	*Chaetoceros tenuissimus* RNA virus *01*
Rename taxon	Species	*Bacillarnavirus nagasakii*	*Rhizosolenia setigera* RNA virus *01*
Rename taxon	Species	*Kusarnavirus tomaruii*	*Astarnavirus*
Rename taxon	Species	*Labyrnavirus takaoii*	*Aurantiochytrium single-stranded* RNA virus *01*
Rename taxon	Species	*Locarnavirus jerichoensis*	*Jericarnavirus B*
Rename taxon	Species	*Locarnavirus greningerii*	*Sanfarnavirus 1*
Rename taxon	Species	*Locarnavirus derisii*	*Sanfarnavirus 2*
Rename taxon	Species	*Locarnavirus rohweri*	*Sanfarnavirus 3*
Rename taxon	Species	*Marnavirus taichanarum*	*Heterosigma akashiwo* RNA virus
Rename taxon	Species	*Salisharnavirus vlokiae*	*Britarnavirus 1*
Rename taxon	Species	*Salisharnavirus britensis*	*Britarnavirus 4*
Rename taxon	Species	*Salisharnavirus mirandaeae*	*Palmarnavirus 128*
Rename taxon	Species	*Salisharnavirus stewardii*	*Palmarnavirus 473*
Rename taxon	Species	*Sogarnavirus gustavseniae*	*Britarnavirus 2*
Rename taxon	Species	*Sogarnavirus kitsilanoensis*	*Britarnavirus 3*
Rename taxon	Species	*Sogarnavirus tomaruii*	*Chaetarnavirus 2*
Rename taxon	Species	*Sogarnavirus kimuraei*	*Chaetenuissarnavirus II*
Rename taxon	Species	*Sogarnavirus culleyi*	*Jericarnavirus A*
Rename taxon	Species	*Sogarnavirus palmerensis*	*Palmarnavirus 156*

*Source/full text: https://ictv.global/ictv/proposals/2024.002F.Marnaviridae_spren.zip

### 2024.003F.Splipalmiviridae_newfam

**Title:** Create 1 new family, including 3 new genera and 16 new species, in the order *Wolframvirales* (class *Amabiliviricetes*, phylum *Lenarviricota*, kingdom *Orthornavirae* and realm *Riboviria*)

**Authors:** Sato Y, Daghino S, Chiba Y, Urayama S, Xie J, Ayllón MA, Suzuki N, Turina M (massimo.turina@ipsp.cnr.it)

**Summary**

**Taxonomic rank(s) affected**
Family, genus and species
**Description of current taxonomy**
Currently unclassified
**Proposed taxonomic change(s)**
We propose to create a new family *Splipalmiviridae*, including 3 new genera which collectively accommodate 16 new species, in the order *Wolframvirales.*
**Justification**
The order *Wolframvirales* currently includes one family *Narnaviridae.* Members of the *family Narnaviridae* have non-segmented positive-sense RNA genomes, each encoding an RdRP. Recently found unclassified splipalmiviruses are phylogenetically close to narnavirids but carry divided RdRPs encoded by two independent genomic segments. Considering the phylogenetic proximity but the different RdRP-encoding strategy compared to narnavirids, we propose to create the new family *Splipalmiviridae* for splipalmiviruses, in the order *Wolframvirales.*
**Submitted**: 20/06/24

**Table 3.** *Splipalmiviridae*, 20 new taxa*

**Table:** 

Operation	Rank	New taxon name	Exemplar	Accession
New taxon	Family	*Splipalmiviridae*		
New taxon	Genus	*Jakapalmivirus*		
New taxon	Species	*Jakapalmivirus sclerotiniae*	Botrytis cinerea binarnavirus 5	RNA1: MN619799; RNA2: MT711187
New taxon	Species	*Jakapalmivirus bremiae*	Bremia lactucae associated splipalmivirus 1	RNA1: MN565689; RNA2: MZ926717; RNA3: OR060921
New taxon	Species	*Jakapalmivirus cinereae*	Botrytis cinerea binarnavirus 1	RNA1: MN619795; RNA2: MT711186
New taxon	Species	*Jakapalmivirus botritidis*	Botrytis cinerea binarnavirus 2	RNA1: MN619796; RNA2: MT119676
New taxon	Species	*Jakapalmivirus ibericum*	downy mildew lesion associated splipalmivirus 3	RNA1: MN539820; RNA2: OQ980200; RNA3: OQ980201
New taxon	Species	*Jakapalmivirus italiense*	downy mildew lesion associated splipalmivirus 4	RNA1: MN539821; RNA2: OQ980202; RNA3: OQ980203
New taxon	Genus	*Divipalmivirus*		
New taxon	Species	*Divipalmivirus italiense*	downy mildew lesion associated splipalmivirus 7	RNA1: MN539824; RNA2: OQ990757
New taxon	Species	*Divipalmivirus aspergilli*	Aspergillus fumigatus narnavirus 2	RNA1: LC553684; RNA2: LC553685; RNA3: LC553686
New taxon	Species	*Divipalmivirus cryphonectriae*	Cryphonectria naterciae splipalmivirus 1	RNA1: LC634419; RNA2: LC634420; RNA3: LC634421; RNA4: LC649880
New taxon	Species	*Divipalmivirus diplodiae*	Diplodia seriata splipalmivirus 1	RNA1: OM837803; RNA2: OM837804; RNA3: OM837805
New taxon	Species	*Divipalmivirus suilli*	Suillus luteus narnavirus 4	RNA1: OQ862540; RNA2: OQ862539
New taxon	Species	*Divipalmivirus japonicum*	Aspergillus flavus narnavirus 1	RNA1: LC763252; RNA2: LC763253; RNA3: LC763254; RNA4: LC763255
New taxon	Genus	*Delepalmivirus*		
New taxon	Species	*Delepalmivirus ibericum*	downy mildew lesion associated splipalmivirus 20	RNA1: MN539837; RNA2: OQ990758; RNA3: OQ990759
New taxon	Species	*Delepalmivirus oidiodendri*	Oidiodendron maius splipalmivirus 1	RNA1: MN736964; RNA2: MN736965; RNA3: MW988098
New taxon	Species	*Delepalmivirus magnaporthae*	Magnaporthe oryzae narnavirus 1	RNA1: LC553711; RNA2: LC553710
New taxon	Species	*Delepalmivirus sclerotiniae*	Sclerotinia sclerotiorum narnavirus 5	RNA1: OK573450; RNA2: OK573451

*Source/full text: https://ictv.global/ictv/proposals/2024.003F.Splipalmiviridae_newfam.zip

### 2024.004F.Oomyviridae_newfam

**Title:** Create a new order, *Lineavirales*, and a new family, *Oomyviridae*, with 3 genera and 38 species in the class *Arfiviricetes* of the phylum *Cressdnaviricota*

**Authors:** Canuti M (marta.canuti@gmail.com), Pénzes J (judit.penzes@tamu.edu)

**Summary**

In 2013, a virus was discovered that was considered to be a ‘hybrid’ between a parvovirus and a circovirus (‘parvovirus-like’ hybrid virus). With the increased use of metagenomics, several recent publications have described similar viruses, proposing their classification and erroneously labelling them in GenBank as parvoviruses. This misclassification issue is continuously increasing and is in dire need of being rectified. Here, we show that these viruses comprise a distinct linear ssDNA virus family (*Oomyviridae*) within the *Cressdnaviricota* and that their unique features and phylogenetic relationships with other members of the class *Arfiviricetes* are strong reasons to include these viruses in a distinct order, for which we propose the name *Lineavirales*, owing to their linear genome. We also show that, although most of these viruses were identified in samples collected from animals, their likely hosts are organisms of the eukaryotic clade Stramenopiles (SAR supergroup).
**Submitted**: 09/06/24; **Revised**: 28/10/24

**Table 4.** *Oomyviridae*, 43 new taxa*. Table too large, see supplementary information sheet supp_info_tab_4

*Source / full text: https://ictv.global/ictv/proposals/2024.004F.Oomyviridae_newfam.zip

### 2024.005F.Pimascovirales_reorg

**Title:** Creation of a new suborder within the *Pimascovirales* to position and name pithovirus-related isolates

**Authors:** Claverie JM (Claverie@igs.cnrs-mrs.fr), Legendre M, Rigou S, Abergel C

**Summary**

**Taxonomic rank(s) affected**
A new suborder, the *Ocovirineae* within the *Pimascovirales*, 3 distinct families: *Pithoviridae*, *Orpheoviridae* and *Hydriviridae.* One family, the *Cedratviridae*, demoted as the new *Orthocedratvirinae* subfamily. Two subfamilies: *Orthopithovirinae* and *Orthocedratvirinae* splitting the *Pithoviridae* family
**Description of current taxonomy**
Previously proposed in proposal #2023.011F by Abrahão and colleagues: two different families: *Pithoviridae* and *Cedratviridae* within the order *Pimascovirales*
**Proposed taxonomic change(s)**
A new suborder, the *Ocovirineae* within the *Pimascovirales,* justified by the need to separate them from the other more distant families (*Marseilleviridae*, *Ascoviridae* and *Iridoviridae*) in the same order*.* The creation of three distinct families: *Pithoviridae*, *Orpheoviridae* and *Hydriviridae* to acknowledge their large differences in genome sizes and gene contents (and accommodate new isolates). The split of the *Pithoviridae* into two subfamilies*: Orthopithovirinae* and *Orthocedratvirinae* to acknowledge their closer proximity compared to members of the other families listed above.
**Justification**
See above
**Submitted**: 13/03/24; **Revised**: 09/10/24

**Table 5.** *Pimascovirales*, five new taxa*

**Table:** 

Operation	Rank	New taxon name	Exemplar	Accession
New taxon	Suborder	*Ocovirineae*		
New taxon	Subfamily	*Orthopithovirinae*		
New taxon	Family	*Hydriviridae*		
New taxon	Genus	*Alphahydrivirus*		
New taxon	Species	*Alphahydrivirus permafrostis*	Siberian hydrivirus MAG1 (R_bin116_k1)	OW988864

**Table 6.** *Pimascovirales*, three move taxa*

**Table:** 

Operation	Rank	Taxon name	Old parent taxon	New parent taxon
Move taxon	Family	*Pithoviridae*	*Pimascovirales*	*Ocovirineae*
Move taxon	Family	*Orpheoviridae*	*Pimascovirales*	*Ocovirineae*
Move taxon	Genus	*Alphapithovirus*	*Pithoviridae*	*Orthopithovirinae*

**Table 7.** *Pimascovirales*, two rename taxa*

**Table:** 

Operation	Rank	New taxon name	Previous taxon name
Rename taxon	Species	*Alphacedratvirus aljazairmassiliense*	*Alphacedratvirus aljazairense*
Rename taxon	Species	*Alphacedratvirus francolausannense*	*Alphacedratvirus franciense*

**Table 8.** *Pimascovirales*, one demote taxon*

**Table:** 

Operation	Old rank (name)	New rank (name)
Demote taxon	Family (*Cedratviridae*)	Subfamily (*Orthocedratvirinae*)

*Source/full text: https://ictv.global/ictv/proposals/2024.005F.Pimascovirales_reorg.zip

### 2024.006F.Amalgaviridae_newgen

**Title:** Create a new genus *Unirnavirus* to accommodate 13 new species within family *Amalgaviridae*

**Authors:** Kotta-Loizou I (i.kotta-loizou13@imperial.ac.uk), Coutts RHA

**Summary**

**Taxonomic rank(s) affected**
Family *Amalgaviridae*
**Description of current taxonomy**
Family *Amalgaviridae* accommodates two genera, *Amalgavirus* and *Zybavirus*
**Proposed taxonomic change(s)**
Within family *Amalgaviridae*, establishing a new genus *Unirnavirus* to accommodate 13 new species
**Justification**
Sequence demarcation and phylogenetic analysis, genome organization and host range
**Submitted**: 20/06/24

**Table 9.** *Amalgaviridae*, 14 new taxa*

**Table:** 

Operation	Rank	*New taxon name*	Exemplar	Accession
New taxon	Genus	*Unirnavirus*		
New taxon	Species	*Unirnavirus aldianthicolae*	Alternaria dianthicola dsRNA virus 1	MT241326
New taxon	Species	*Unirnavirus allongipei*	Alternaria longipes non-segmented mycovirus 1	KJ817371
New taxon	Species	*Unirnavirus aspergilli*	Aspergillus lentulus non-segmented dsRNA virus 1	LC553704
New taxon	Species	*Unirnavirus beauveriae*	Beauveria bassiana non-segmented RNA virus 1	LN610699
New taxon	Species	*Unirnavirus cogleosporioidei*	Colletotrichum gloeosporioides RNA virus 1	ON887156
New taxon	Species	*Unirnavirus cohigginsiani*	Colletotrichum higginsianum non-segmented dsRNA virus 1	KM923925
New taxon	Species	*Unirnavirus combuense*	Combu double-strand RNA mycovirus	MH990637
New taxon	Species	*Unirnavirus fusarii*	Fusarium culmorum virus 1	MN187541
New taxon	Species	*Unirnavirus pripenicillii*	Penicillium janczewskii Beauveria bassiana-like virus 1	KT601106
New taxon	Species	*Unirnavirus prustilaginoideae*	Ustilaginoidea virens unassigned RNA virus HNND 1	KR106133
New taxon	Species	*Unirnavirus secupenicillii*	Penicillium citrinum non-segmented RNA virus 1	OP103962
New taxon	Species	*Unirnavirus secustilaginoideae*	Ustilaginoidea virens RNA virus M-A	ON791647
New taxon	Species	*Unirnavirus trichodermae*	Trichoderma harzianum mycovirus 1	MH155602

*Source/full text: https://ictv.global/ictv/proposals/2024.006F.Amalgaviridae_newgen.zip

### 2024.007F.Mycoalphaviridae_newfam

**Title:** Create one new family (*Mycoalphaviridae*) including two new genera (*Alphasclernavirus* and *Betasclernavirus*) and seven new species

**Authors:** Xie J (jiataoxie@mail.hzau.edu.cn), Mu F, Jia J, Jiang D, Sabanadzovic S

**Summary**

**Taxonomic rank(s) affected**
*Hepelivirales*
**Description of current taxonomy**
The order including 4 families and 27 species.
**Proposed taxonomic change(s)**
Create one new family (*Mycoalphaviridae*) including two new genera (*Alphasclernavirus* and *Betasclernavirus*) and seven new species.
**Justification**
Members in the proposed family *Mycoalphaviridae* have a single-stranded positive-sense RNA genome of 6.0 to 10.1 kb with one or more ORFs. Members of the proposed family have only been identified in fungi and oomycetes. The RNA-directed RNA polymerase (RdRP) of viruses in the family *Mycoalphaviridae* has the closest similarity to viruses of the order *Hepelivirales*, though the identity is lower than 20%. These low-level amino acid sequence identities, the different host ranges and the result of phylogenetic analysis both support the establishment of the new family. The proposed family *Mycoalphaviridae* includes two proposed genera *Alphasclernavirus* and *Betasclernavirus* that accommodate three and seven species, respectively. The RdRP amino acid sequence identity between members of different genera and between members of different species is lower than 26% and 50%, respectively, in the family.
**Submitted**: 19/05/24 ; **Revised**: 18/10/24

**Table 10.** *Mycoalphaviridae*, 10 new taxa*

**Table:** 

Operation	Rank	New taxon name	Exemplar	Accession
New taxon	Family	*Mycoalphaviridae*		
New taxon	Genus	*Alphasclernavirus*		
New taxon	Species	*Alphasclernavirus alphasclerotiniae*	Sclerotinia sclerotiorum mycoalphavirus virus 1	MT706025
New taxon	Species	*Alphasclernavirus betasclerotiniae*	Sclerotinia sclerotiorum RNA virus L	EU779934
New taxon	Genus	*Betasclernavirus*		
New taxon	Species	*Betasclernavirus alphafusarii*	Fusarium graminearum alphavirus-like virus 1	MN400076
New taxon	Species	*Betasclernavirus botrytidis*	Botrytis cinerea alpha-like virus 1	MN625250
New taxon	Species	*Betasclernavirus betafusarii*	Fusarium sacchari alphavirus-like virus 1	MN295968
New taxon	Species	*Betasclernavirus betasclerotii*	Sclerotium rolfsii alphavirus-like virus 1	MH766488
New taxon	Species	*Betasclernavirus alphasclerotii*	Sclerotium rolfsii alphavirus-like virus 3	MH766490

*Source/full text: https://ictv.global/ictv/proposals/2024.007F.Mycoalphaviridae_newfam.zip

### 2024.008F.Orpoviricetes_newclass

**Title:** Create a new class, *Orpoviricetes,* including two new orders, four families, seven genera and 26 new species in the kingdom *Orthornavirae* (realm *Riboviria*)

**Authors:** Botella L, Turina M, Hejna O, Krupovic M, Neri U, Poimala A, Shamsi W, Sabanadzovic S, Sutela S, Vainio E, Forgia M (marco.forgia@ipsp.cnr.it)

**Summary**

**Taxonomic rank(s) affected**
*Riboviria* and *Orthornavirae*
**Description of current taxonomy**
The kingdom *Orthornavirae* includes six phyla which were established based on phylogenetic analysis of the RdRP and comparative analysis of the viral genomes and encoded proteins.
**Proposed taxonomic change(s)**
Creation of a new class *Orpoviricetes*, 2 new orders, 4 families and 7 genera which collectively accommodate 26 new species for ormycoviruses, recently identified RNA viruses that infect fungi and oomycetes. These viruses have genomes that consist of two monocistronic ssRNA segments, with RNA1 encoding a putative RdRP and RNA2 encoding a hypothetical protein with an unknown function.
**Justification**
Viruses from the kingdom *Orthornavirae*, which encompasses RNA viruses that encode RNA-directed RNA polymerases (RdRPs), generally have a highly conserved motif C. This motif, often containing the core amino acid triplet GDD, is critical for the catalytic activity of the RdRP. Other triplets more rarely occurring are NDD, SDD, GDN, IDD, ADN and ADD (in order of frequency; [18]). However, ormycoviruses exhibit unique variations in the core amino acid triad of motif C (e.g. NDD, GDQ and HDD) not found in other RNA viruses. Based on the significant variations in the conserved motif C and the high divergence from other RNA viruses (not conserved enough to be retrieved by blast searches using any of the RdRP encoded by viruses classified in the six currently recognized phyla), there is a strong case for considering ormycoviruses as members of, at least, a distinct class. Variations within the C motifs are rare but not unprecedented in other RNA viruses, so there is still a need to carry out phylogenetic and structural analyses to confirm whether ormycoviruses have diverged from viruses within existing phyla or have diverged prior to the radiation of viruses classified in the six currently established phyla. Therefore, as an initial step in the official classification of these viruses, we propose to classify them within a new class not assigned to an existing phylum within the kingdom *Orthornavirae.* This classification would reflect their unique evolutionary pathway and potentially distinct biological characteristics.
**Submitted**: 20/06/24; **Revised**: 17/12/24

**Table 11.** *Orpoviricetes*, 40 new taxa*

**Table:** 

Operation	Rank	New taxon name	Exemplar	Accession
New taxon	Class	*Orpoviricetes*		
New taxon	Order	*Formycovirales*		
New taxon	Family	*Gammaormycoviridae*		
New taxon	Genus	*Hormycovirus*		
New taxon	Species	*Hormycovirus hortiboleti*	Hortiboletus rubellus ormycovirus 1	RNA1: PP260025; RNA2: PP260026
New taxon	Genus	*Tormycovirus*		
New taxon	Species	*Tormycovirus erysiphe*	Erysiphe lesion associated ormycovirus 4	RNA1: OM272933; RNA2: OM272934
New taxon	Species	*Tormycovirus thrichodermae*	Trichoderma tomentosum ormycovirus 1	RNA1: OQ463855; RNA2: OQ463856
New taxon	Species	*Tormycovirus fusarii*	Fusarium graminearum ormycovirus 1	RNA1: PP658032; RNA2: PP658033
New taxon	Species	*Tormycovirus unplasmoparae*	downy mildew lesion associated ormycovirus 4	RNA1: OM272935; RNA2: OM272936
New taxon	Species	*Tormycovirus duaplasmoparae*	downy mildew lesion associated ormycovirus 5	RNA1: OM272937; RNA2: OM272938
New taxon	Family	*Betaormycoviridae*		
New taxon	Genus	*Vormycovirus*		
New taxon	Species	*Vormycovirus unerysiphe*	Erysiphe lesion associated ormycovirus 2	RNA1: OM272931; RNA2: OM272932
New taxon	Species	*Vormycovirus duerysiphe*	Erysiphe lesion associated ormycovirus 3	RNA1: OM363731; RNA2: OM363732
New taxon	Species	*Vormycovirus plasmoparae*	downy mildew lesion associated ormycovirus 3	RNA1: OM363729; RNA2: OM363730
New taxon	Species	*Vormycovirus verticilli*	Verticillium dahliae ormycovirus 2	RNA1: OR734292; RNA2: OR734293
New taxon	Species	*Vormycovirus ophiocordyceps*	Ophiocordyceps sinensis ormycovirus 1	RNA1: PP623130; RNA2: PP623131
New taxon	Genus	*Stormycovirus*		
New taxon	Species	*Stormycovirus starmellariae*	Starmerella bacillaris ormycovirus 1	RNA1: OM272929; RNA2: OM272930
New taxon	Species	*Stormycovirus alariae*	Alaria esculenta RNA virus 1	RNA1: PP793779; RNA2: PP793780
New taxon	Order	*Bormycovirales*		
New taxon	Family	*Alphaormycoviridae*		
New taxon	Genus	*Phormycovirus*		
New taxon	Species	*Phormycovirus phytophthorae*	Phytophthora cinnamomi ormycovirus 7–5	RNA1: PP891879; RNA2: PP891862
New taxon	Species	*Phormycovirus unphytophthorae*	Phytophthora cinnamomi ormycovirus 4–1	RNA1: PP891842; RNA2: PP891839
New taxon	Species	*Phormycovirus duphytophthorae*	Phytophthora cinnamomi ormycovirus 5–2	RNA1: PP891849; RNA2: PP891846
New taxon	Species	*Phormycovirus trephytophthorae*	Phytophthora cinnamomi ormycovirus 6–4	RNA1: PP891858; RNA2: PP891851
New taxon	Species	*Phormycovirus quaphytophthorae*	Phytophthora cinnamomi ormycovirus 11–3	RNA1: PP891940; RNA2: PP891934
New taxon	Species	*Phormycovirus plasmoparae*	downy mildew lesion associated ormycovirus 2	RNA1: OM262448; RNA2: PP940184
New taxon	Genus	*Dormycovirus*		
New taxon	Species	*Dormycovirus erysiphe*	Erysiphe lesion associated ormycovirus 1	RNA1: OM272927; RNA2: OM272928
New taxon	Species	*Dormycovirus plasmoparae*	downy mildew lesion associated ormycovirus 1	RNA1: OM363727; RNA2: OM363728
New taxon	Species	*Dormycovirus phytophthorae*	Phytophthora cinnamomi ormycovirus 9–16	RNA1: PP891926; RNA2: PP891910
New taxon	Family	*Deltanormycoviridae*		
New taxon	Genus	*Bormycovirus*		
New taxon	Species	*Bormycovirus verticilli*	Verticillium dahliae ormycovirus 1	RNA1: OR734290; RNA2: OR734291
New taxon	Species	*Bormycovirus unphytophthorae*	Phytophthora cinnamomi ormycovirus 1–1	RNA1: PP891751; RNA2: PP891713
New taxon	Species	*Bormycovirus duphytophthorae*	Phytophthora cinnamomi ormycovirus 2–25	RNA1: PP891801; RNA2: PP891774
New taxon	Species	*Bormycovirus trephytophthorae*	Phytophthora cinnamomi ormycovirus 3–7	RNA1: PP891825; RNA2: PP891808

*Source/full text: https://ictv.global/ictv/proposals/2024.008F.Orpoviricetes_newclass.zip

## Supplementary material

10.1099/jgv.0.002115 Uncited Supplementary Material 1.

## Abbreviations

ICTV: International Committee on Taxonomy of Viruses
RdRP: RNA-directed RNA polymerase
SC: Subcommittee
SG: Study Groups
