# Erratum: Summary of taxonomy changes ratified by the International Committee on Taxonomy of Viruses (ICTV) from the Fungal and Protist Viruses Subcommittee, 2025

**DOI:** 10.1099/jgv.0.002144

**Published:** 2025-08-13

**Authors:** Sead Sabanadzovic, Chantal Abergel, María A. Ayllón, Leticia Botella, Marta Canuti, Yuto Chiba, JeanMichel Claverie, Robert H.A Coutts, Stefania Daghino, Livia Donaire, Marco Forgia, Ondřej Hejna, Jichun Jia, Daohong Jiang, Ioly Kotta-Loizou, Mart Krupovic, Andrew S. Lang, Matthieu Legendre, Shin-Yi Lee Marzano, Fan Mu, Uri Neri, Luca Nerva, Judit Pénzes, Anna Poimala, Sofia Rigou, Yukiyo Sato, Wajeeha Shamsi, Suvi Sutela, Nobuhiro Suzuki, Massimo Turina, Syun-ichi Urayama, Eeva J. Vainio, Jiatao Xie, ICTV Taxonomy Summary Consortium

**Affiliations:** 1 Department of Agricultural Science and Plant Protection, Mississippi State University, Starkville, MS, USA; 2 Institute for Genomics, Biocomputing and Biotechnology, Mississippi State University, Starkville, MS, USA; 3 Information Génomique & Structurale, UMR7256, CNRS & Aix-Marseille Université, Marseille, IMM, IM2B, IOM,, Marseille, France; 4 Centro de Biotecnología y Genómica de Plantas, Universidad Politécnica de Madrid (UPM)— Instituto Nacional de Investigación y Tecnología Agraria y Alimentaria (INIA/CSIC), Campus de Montegancedo, Pozuelo de Alarcón, Madrid, Spain; 5 Departamento de Biotecnología-Biología Vegetal, Escuela Técnica Superior de Ingeniería Agronómica, Alimentaria y de Biosistemas, Universidad Politécnica de Madrid (UPM), Madrid, Spain; 6 Forest Protection and Wildlife Management Mendel University in Brno, Brno, Czechia; 7 Department of Veterinary and Animal Sciences, University of Copenhagen, Frederiksberg, Denmark; 8 School of Agriculture, Meiji University, Kawasaki, Japan; 9 School of Health, Medicine and Life Sciences, University of Hertfordshire, Hatfield, UK; 10 Institute for Sustainable Plant Protection, National Research Council of Italy, Torino, Italy; 11 Centro de Edafología y Biología Aplicada del Segura-CSIC, 30100 Murcia, Spain; 12 Institute for Sustainable Plant Protection, CNR, Bari, Italy; 13 Department of Genetics and Biotechnologies, University of South Bohemia, České Budějovice, Czechia; 14 College of Plant Protection, Shanxi Agricultural University, Jinzhong, Shanxi, PR China; 15 College of Plant Science and Technology, Huazhong Agricultural University, Wuhan, PR China; 16 Institut Pasteur, Université Paris Cité, CNRS UMR6047, Archaeal Virology Unit, Paris, France; 17 Department of Biology, Memorial University of Newfoundland, St. John’s, Canada; 18 United States Department of Agriculture, Agricultural Research Service, Application Technology Research Unit, Toledo, Ohio, USA; 19 The Shmunis School of Biomedicine and Cancer Research, Tel Aviv University, Tel Aviv, Israel; 20 Council for Agricultural Research and Economics - Research Centre for Viticulture and Enology, Roma, Italy; 21 Department of Entomology, Texas A&M University, College Station, TX, USA; 22 Natural Resources Institute Finland (Luke), Helsinki, Finland; 23 Department of Biology, Institute for Plant Sciences, University of Cologne, Cologne, Germany; 24 Department of Molecular Biology and Genetics, Aarhus University, 8000 Aarhus C, Denmark; 25 Institute of Plant Science and Resources, Okayama University, Kurashiki, Japan; 26 Institute for Sustainable Plant Protection-URT Brescia, National Research Council of Italy, 25123, Brescia, Italy; 27 Department of Plant Protection, School of Agriculture, The University of Jordan, Amman, 11942, Jordan; 28 Department of Life and Environmental Sciences, University of Tsukuba, Tsukuba, Japan

In the published version of this article, there were three authors omitted from the author list.

These were, Fan Mu, College of Plant Protection, Shanxi Agricultural University, Jinzhong, Shanxi, PR China; Uri Neri, The Shmunis School of Biomedicine and Cancer Research, Tel Aviv University, Israel; Suvi Sutela, Natural Resources Institute Finland (Luke), Helsinki, Finland.

Previously, the author and affiliation list appeared as below:

Sead Sabanadzovic^1,2, *^, Chantal Abergel^3^, María A. Ayllón^4,5^, Leticia Botella^6^, Marta Canuti^7^, Yuto Chiba^8^, JeanMichel Claverie^3^, Robert H.A. Coutts^9^, Stefania Daghino^10^, Livia Donaire^11^, Marco Forgia^12^, Ondřej Hejna^13^, Jichun Jia^14^, Daohong Jiang^15^, Ioly Kotta-Loizou^9^, Mart Krupovic^16^, Andrew S. Lang^17^, Matthieu Legendre^3^, Shin-Yi Lee Marzano^18^, Luca Nerva^19^, Judit Pénzes^20^, Anna Poimala^21^, Sofia Rigou^3^, Yukiyo Sato^22^, Wajeeha Shamsi^23^, Nobuhiro Suzuki^24^, Massimo Turina^25,26^, Syun-ichi Urayama^27^, Eeva J. Vainio^21^, Jiatao Xie^15^ and ICTV Taxonomy Summary Consortium

Author affiliations:

^1^Department of Agricultural Science and Plant Protection, Mississippi State University, Mississippi, USA; ^2^Institute for Genomics, Biocomputing and Biotechnology, Mississippi State University, Mississippi, USA; ^3^Information Génomique and Structurale, UMR7256, CNRS and Aix-Marseille Université, Marseille, IMM, IM2B, IOM, Marseille, France; ^4^Centro de Biotecnología y Genómica de Plantas, Universidad Politécnica de Madrid (UPM)— Instituto Nacional de Investigación y Tecnología Agraria y Alimentaria (INIA/CSIC), Campus de Montegancedo, Pozuelo de Alarcón, Madrid, Spain; ^5^Departamento de Biotecnología-Biología Vegetal, Escuela Técnica Superior de Ingeniería Agronómica, Alimentaria y de Biosistemas, Universidad Politécnica de Madrid (UPM), Madrid, Spain; ^6^Forest Protection and Wildlife Management Mendel University in Brno, Brno, Czechia; ^7^Department of Veterinary and Animal Sciences, University of Copenhagen, Frederiksberg, Denmark; ^8^School of Agriculture, Meiji University, Kawasaki, Japan; ^9^School of Health, Medicine and Life Sciences, University of Hertfordshire, Hatfield, UK; ^10^Institute for Sustainable Plant Protection, National Research Council of Italy, Torino, Italy; ^11^Centro de Edafología y Biología Aplicada del Segura-CSIC, 30100 Murcia, Spain; ^12^Institute for Sustainable Plant Protection, CNR, Bari, Italy; ^13^Department of Genetics and Biotechnologies, University of South Bohemia, České Budějovice, Czechia; ^14^College of Plant Protection, Shanxi Agricultural University, Jinzhong, Shanxi, PR China; ^15^College of Plant Science and Technology, Huazhong Agricultural University, Wuhan, PR China; ^16^Institut Pasteur, Université Paris Cité, CNRS UMR6047, Archaeal Virology Unit, Paris, France; ^17^Department of Biology, Memorial University of Newfoundland, St. John’s, Canada; ^18^United States Department of Agriculture, Agricultural Research Service, Application Technology Research Unit, Toledo, Ohio, USA; ^19^Council for Agricultural Research and Economics - Research Centre for Viticulture and Enology, Roma, Italy; ^20^Department of Entomology, Texas A and M University, College Station, TX, USA; ^21^Natural Resources Institute Finland (Luke), Helsinki, Finland; ^22^Department of Biology, Institute for Plant Sciences, University of Cologne, Cologne, Germany; ^23^Department of Molecular Biology and Genetics, Aarhus University, 8000 Aarhus C, Denmark; ^24^Institute of Plant Science and Resources, Okayama University, Kurashiki, Japan; ^25^Institute for Sustainable Plant Protection-URT Brescia, National Research Council of Italy, 25123, Brescia, Italy; ^26^Department of Plant Protection, School of Agriculture, The University of Jordan, Amman, 11942, Jordan; ^27^Department of Life and Environmental Sciences, University of Tsukuba, Tsukuba, Japan.

The correct author and affiliation list should have appeared as:

Sead Sabanadzovic^1,2, *^, Chantal Abergel^3^, María A. Ayllón^4,5^, Leticia Botella^6^, Marta Canuti^7^, Yuto Chiba^8^, JeanMichel Claverie^3^, Robert H.A. Coutts^9^, Stefania Daghino^10^, Livia Donaire^11^, Marco Forgia^12^, Ondřej Hejna^13^, Jichun Jia^14^, Daohong Jiang^15^, Ioly Kotta-Loizou^9^, Mart Krupovic^16^, Andrew S. Lang^17^, Matthieu Legendre^3^, Shin-Yi Lee Marzano^18^, Fan Mu^14^, Uri Neri^19^, Luca Nerva^20^, Judit Pénzes^21^, Anna Poimala^22^, Sofia Rigou^3^, Yukiyo Sato^23^, Wajeeha Shamsi^24^, Suvi Sutela^22^, Nobuhiro Suzuki^25^, Massimo Turina^26,27^, Syun-ichi Urayama^28^, Eeva J. Vainio^22^, Jiatao Xie^15^ and ICTV Taxonomy Summary Consortium

Author affiliations:

^1^Department of Agricultural Science and Plant Protection, Mississippi State University, Mississippi, USA; ^2^Institute for Genomics, Biocomputing and Biotechnology, Mississippi State University, Mississippi, USA; ^3^Information Génomique and Structurale, UMR7256, CNRS and Aix-Marseille Université, Marseille, IMM, IM2B, IOM, Marseille, France; ^4^Centro de Biotecnología y Genómica de Plantas, Universidad Politécnica de Madrid (UPM)— Instituto Nacional de Investigación y Tecnología Agraria y Alimentaria (INIA/CSIC), Campus de Montegancedo, Pozuelo de Alarcón, Madrid, Spain; ^5^Departamento de Biotecnología-Biología Vegetal, Escuela Técnica Superior de Ingeniería Agronómica, Alimentaria y de Biosistemas, Universidad Politécnica de Madrid (UPM), Madrid, Spain; ^6^Forest Protection and Wildlife Management Mendel University in Brno, Brno, Czechia; ^7^Department of Veterinary and Animal Sciences, University of Copenhagen, Frederiksberg, Denmark; ^8^School of Agriculture, Meiji University, Kawasaki, Japan; ^9^School of Health, Medicine and Life Sciences, University of Hertfordshire, Hatfield, UK; ^10^Institute for Sustainable Plant Protection, National Research Council of Italy, Torino, Italy; ^11^Centro de Edafología y Biología Aplicada del Segura-CSIC, 30100 Murcia, Spain; ^12^Institute for Sustainable Plant Protection, CNR, Bari, Italy; ^13^Department of Genetics and Biotechnologies, University of South Bohemia, České Budějovice, Czechia; ^14^College of Plant Protection, Shanxi Agricultural University, Jinzhong, Shanxi, PR China; ^15^College of Plant Science and Technology, Huazhong Agricultural University, Wuhan, PR China; ^16^Institut Pasteur, Université Paris Cité, CNRS UMR6047, Archaeal Virology Unit, Paris, France; ^17^Department of Biology, Memorial University of Newfoundland, St. John’s, Canada; ^18^United States Department of Agriculture, Agricultural Research Service, Application Technology Research Unit, Toledo, Ohio, USA; ^19^The Shmunis School of Biomedicine and Cancer Research, Tel Aviv University, Israel; ^20^Council for Agricultural Research and Economics - Research Centre for Viticulture and Enology, Roma, Italy; ^21^Department of Entomology, Texas A and M University, College Station, TX, USA; ^22^Natural Resources Institute Finland (Luke), Helsinki, Finland; ^23^Department of Biology, Institute for Plant Sciences, University of Cologne, Cologne, Germany; ^24^Department of Molecular Biology and Genetics, Aarhus University, 8000 Aarhus C, Denmark; ^25^Institute of Plant Science and Resources, Okayama University, Kurashiki, Japan; ^26^Institute for Sustainable Plant Protection-URT Brescia, National Research Council of Italy, 25123, Brescia, Italy; ^27^Department of Plant Protection, School of Agriculture, The University of Jordan, Amman, 11942, Jordan; ^28^Department of Life and Environmental Sciences, University of Tsukuba, Tsukuba, Japan.

The version of record of this article will be updated to reflect the correction to the author list.

The Microbiology Society apologises for any inconvenience caused.

